# Adult outcome of pediatric hydrocephalus

**DOI:** 10.1007/s00381-012-1723-y

**Published:** 2012-02-19

**Authors:** Matthieu Vinchon, Marc Baroncini, Isabelle Delestret

**Affiliations:** Department of Pediatric Neurosurgery, Hôpital Roger Salengro, Lille University Hospital, CHRU de Lille, Lille, 59037 Cedex, France

**Keywords:** Pediatric hydrocephalus, Mortality, Shunt obstruction, Child-to-adult transition

## Abstract

**Introduction:**

The outlook of pediatric hydrocephalus has spectacularly improved over the past decades; however, the adult outcome is still poorly documented. Determining the healthcare profile of these patients is important in order to organize the management of this growing population. We decided to review our pediatric hydrocephalus database for pediatric patients treated for hydrocephalus and followed up into adulthood.

**Methods:**

Our institution has a virtual monopoly for pediatric hydrocephalus, serving a four-million-plus population; the transition to adult care is also managed in the same institution. We retrospectively reviewed patients younger than 18 treated for hydrocephalus since 1980 and followed up beyond the age of 20.

**Results:**

We reviewed 456 patients, with a mean initial age of 55.6 months, and followed up for a mean of 24.2 years. In 81 patients (17.8%), the last shunt operation occurred after 20 years; 22 of these (4.9% of the total) having never been revised earlier. Sixteen patients (3.5%) could be weaned of their shunt. Thirteen patients died in adult age, 5 of these dying of shunt-related causes. The most prominent sequels were motor (46.5%) and cognitive (47.6%); only 82 patients (18.0%) had no sequel at all. Intelligence quotient values were ≥80 in 54.5% of tested patients; however, schooling was normal in only 41.4%, and only 33.7% was employed in the competitive labor market.

**Conclusion:**

Adults treated for hydrocephalus in childhood require a life-long follow-up. Late mortality is low but not null, morbidity is high, and many patients require shunt surgery during adulthood. The transition from child to adult neurosurgery needs to be organized for these vulnerable patients.

## Introduction

Historically, the outcome of hydrocephalus used to be appalling: Laurence reported in 1962 a 20% survival rate into adulthood, the surviving patients having generally severe sequels [17]. The outcome of hydrocephalic children has undergone a sea change when shunts were introduced in the 1960s, with most patients surviving the initial phase and growing into adulthood. However, the long-term outcome of children treated for hydrocephalus is poorly documented because most studies available were based on surveys, and thus subject to important selection biases, and involving limited numbers of patients [8, 19, 21, 22], or very detailed studies with a short follow-up [16, 24]. In our hospital, we have organized a long time ago a systematic follow-up of pediatric patients in the adult neurosurgery clinic when they become adults.

We decided to review our clinical database in order to study the outcome of patients treated for hydrocephalus during childhood and followed up beyond their 20th birthday.

## Material and methods

Our hospital is the sole referral center for pediatric neurosurgery, serving a four-million population area. Our patients have been logged prospectively in a database since the 1970s, which became computerized in the 1990s. The patients were followed up systematically in pediatric neurosurgical clinics. Our policy it to follow-up patients every second year for life, and systematic appointments are sent from our secretariat; once the patients become adults (around 20 years), the biannual neurosurgical follow-up is continued in the adult neurosurgical clinic. Our policy is to revise asymptomatic ruptured shunts whenever the patient is considered shunt-dependent, which is attested by a previous history of symptomatic shunt failure or a positive shuntogram (patent shunt) or badly tolerated ligation test [31].

We retrospectively selected cases of patients <18 years operated for hydrocephalus between January 1980 and December 2007 and followed up beyond the age of 20. Reoperations included all surgical procedures including test ligation and removal; shunt infection was defined as a clinical diagnosis of infection, with or without positive culture, occurring any time during the follow-up, leading either to shunt removal or the patient’s demise; shunt failure was defined as shunt malfunction or infection requiring reoperation or leading to the patient’s demise. Functional outcome was evaluated quantitatively using the Karnofsky independence scale (100 = asymptomatic, 80 = able to assume normal life, 0 = dead) and the Glasgow Outcome Score (GOS) according to the World Federation of Neurosurgical Societies (1 = normal life, 5 = dead) and qualitatively by rating the different sequels as present or absent. Intelligence quotient (IQ) evaluation was performed using the WISC III test in patients selected on a case-by-case basis; for the purpose of the study, all results for a given patient were averaged; normal IQ was defined as an average IQ at or above 80. Schooling was rated as: normal curriculum; shortened curriculum in a normal school; schooling with aides (like support during classes, additional time for exams, etc.); school in specialized institution; and none at all. Social status at last control was rated as: normal job (full employment in the competitive labor market or autonomous mother-at-home); unemployed (job seeker on the competitive labor market); sheltered job (granted or in waiting); handicapped (living on a pension at home or in a shelter); whenever the young adult had not completed his learning curriculum (whichever it was), the case was rated as “non-evaluable.” The unemployment rate was defined as the ratio job seeker/(normal job + job seekers).

As the study was mostly retrospective, we used only descriptive statistics and no tests; actuarial survival was calculated using the Kaplan–Meier method, using the software SPSS 15.0 for Windows.

## Results

### Constitution of the series

During the period of the study, 1,973 patients were followed up for hydrocephalus; among these, 274 died before reaching the age of 20 years and 1,243 were either under 20 or lost to follow-up at the time of the collection of data. The remaining 456 patients represent the study group. These patients were 236 male and 220 female, the median age at first shunt implantation was 11.0 months (mean, 55.6; 95% CI, 54.3–57.0); only 50 were more than 15 years old at the time of initial treatment. The causes of hydrocephalus were tumor in 125 (27.4%), spina bifida in 85 (18.6%), meningitis in 59 (12.9%), hemorrhage in 57 (12.5%), arachnoid cyst in 32 (7.0%), other cerebral malformations in 55 (12.1%), traumatic in 15 (3.3%), Dandy–Walker syndrome in 6 (1.3%), other skeletal malformation in 5 (1.1%), and unknown in 17 (3.7%). The initial treatments were ventriculoperitoneal shunt in 376 (82.5%), endoscopic procedure in 40 (8.8%), and other shunts in 40 (8.8%). The median follow-up was 23.0 years (range, 20 to 44; mean, 24.2; 95% CI, 24.0–24.3).

### Surgical outcome

**Fig. 1:**
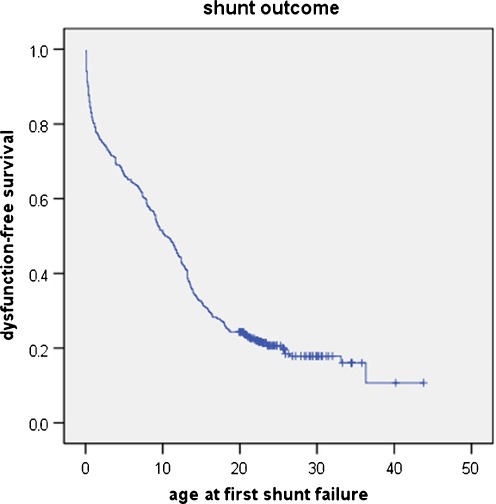
Actuarial event-free survival after the first surgery. This curve shows the characteristic biphasic curve initially, and then the curve shows a shoulder around 15 years, becoming almost parallel to the baseline. However, several patients had their first shunt revision after 20 years, showing that patients who have never been revised before cannot be considered as shunt-independent

**Fig. 2:**
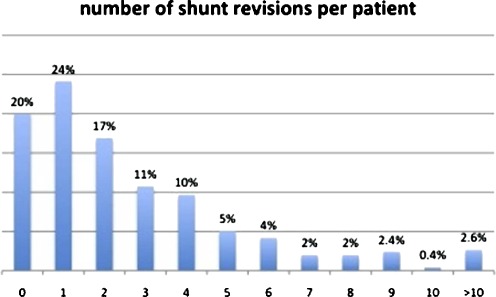
Number of reoperations per patient. Most patients had at least one reoperation, although 91 (20.0%) had not required any reoperation at last control

During the period of follow-up, these 456 patients underwent 1,226 reoperations (mean, 2.7 per patient). The surgical outcome after initial surgery is shown in Fig. 1, and the number of reoperations per patient is detailed in Fig. 2. Overall, 91 patients had not been reoperated yet at the last control, 346 had at least one reoperation before reaching the age of 20, and 81 (17.8% of the series) underwent at least one reoperation after the age of 20; in 22 of these (4.9% of the series), the first reoperation took place after the age of 20. Five patients in the series died of shunt-related causes after the age of 20; in one of these, the diagnosis of shunt failure was delayed in part because the shunt had never been revised before. Over the whole series, the shunt-related mortality rate could be established at 4.2% before the age of 20 years and 7.2% at 30 years.

Shunt infection occurred 88 times in 71 patients (5.2% per operation, 15.6% per patient); in 11 patients, shunt infection occurred after the age of 20, leading to death in one case. Procedures aimed at achieving shunt independence were attempted in 27 cases: through shuntography and/or ligature (15), endoscopy (7), or forced removal because of infection (5); this resulted in shunt independence in 16 and failure to achieve shunt independence in 11. Latex allergy was recorded in 36 cases (7.9%), associated with myelomeningocele in 25 cases (representing 29.4% of patients with myelomeningocele).

### Mortality

**Fig. 3:**
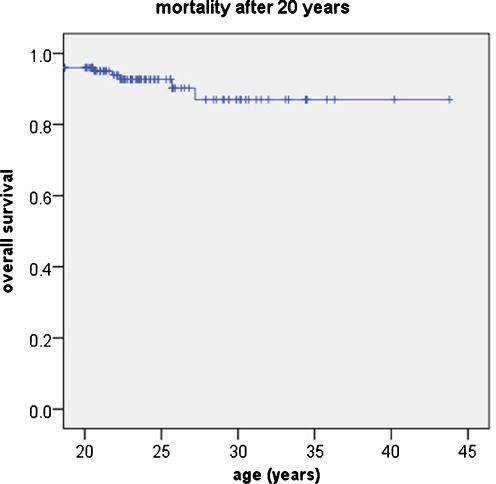
Mortality after the age of 20. Thirteen patients died between 20 and 27 years, the actuarial mortality was 13%

As mentioned earlier, mortality before 20 years affected 287 patients, making for an actuarial mortality rate of 18.1% at 20 years. Among survivors, 15 (2.85%) died after the age of 20 years, with 5 of these dying of shunt-related causes. The last casualty was recorded at the age of 27 years; the actuarial overall mortality rate after 20 years was 6.3% (Fig. 3).

### Functional outcome

**Fig. 4:**
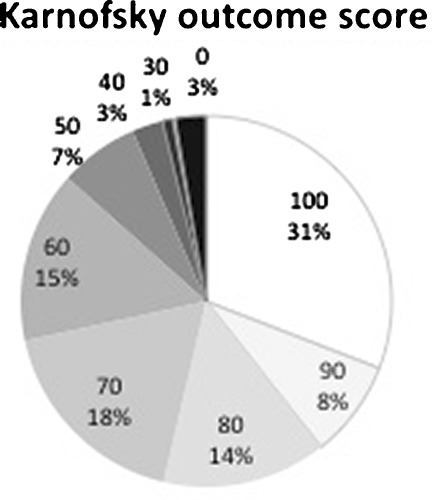
Distribution of Karnofsky independence score; 53.8% had a KNK score at or above 80 (independent life)

**Fig. 5:**
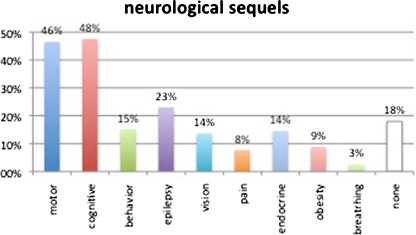
Prevalence of the different sequels among the patients of the series. Only 82 patients (18.0%) were free of all of these ailments

Karnofsky independence scale ratings were available in 448 patients (98.2%); the results are detailed in Fig. 4; overall, 241 (53.7%) had a KNK score at or above 80, meaning autonomous activity. The most common sequels were cognitive in 217 (47.6%), motor in 212 (46.6%), epilepsy in 105 (23.0%), behavior disturbance in 69 (15.1%), endocrine disorders in 66 (14.5%), vision loss in 62 (13.6%), pain in 35 (14.5%), and breathing problems in 12 (2.6%) cases. In actuarial analysis, the cumulated incidence of secondary epilepsy was 6.1% at 20 years and 13.1% at 30 years. Only 82 patients (18.0%) were immune to any of these ailments at last control (Fig. 5). Pregnancy was recorded in 32 cases (14.5% of female patients).

**Fig. 6:**
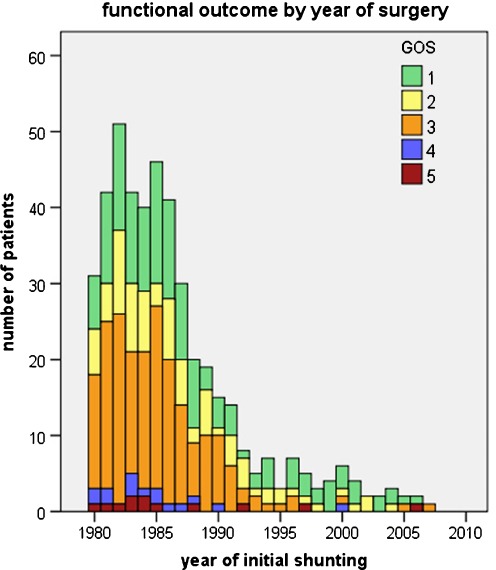
Overall outcome. The outcome was evaluated with the GOS (GOS 1 = normal life, GOS 5 = dead) according to the year of initial treatment; this figure suggests that mortality and severe morbidity (GOS 3 to 5) were higher for patients treated in the 1980s and early 1990s. However, patients treated in the 1990s must have been older than the average at the time of initial treatment to meet the inclusion criteria, and their follow-up was also shorter, introducing important biases

The functional outcome by cause of hydrocephalus is detailed in Table 1; our data show a generally poorer outcome for hydrocephalus diagnosed at birth (malformative, posthemorrhagic, postmeningitis, and myelomeningocele), compared with other causes like arachnoid cysts or tumoral hydrocephalus. We also studied the overall outcome (evaluated with the GOS) according to the year of initial treatment, plotted in Fig. 6; this figure suggests that mortality and severe morbidity (GOS 3 to 5) were higher for patients treated in the 1980s and early 1990s.

**Table 1:** Clinical outcome in the total series and in subgroups by cause of hydrocephalus

	Number of patients	Mean age at first surgery (months)	Reoperations (mean per patient)	Infections (prevalence per patient)	Mortality (cause)	Mean Karnofsky	Good outcome (GOS 1)	No sequels	Motor sequels	Epilepsy	Cognitive sequels	IQ ≥ 80 (number tested)	Median IQ (extremes)	Normal schooling	Normal job (percent of evaluable^a^ cases)
Tumor	125	108.6	1.8	13 (10.4%)	6 (5 tumor, 1 shunt)	80.6	43 (34.4%)	22 (17.6%)	37 (29.6%)	28 (22.4%)	61 (48.8%)	55.6% (72)	82.4 (48–123)	64 (51.2%)	44 (45.4%)
Myelomeningocele	85	33.0	2.6	13 (15.3%)	3 (2 shunt, 1 intercurrent)	65.1	1 (1.2%)	0	84 (98.8%)	9 (10.6%)	32 (37.6%)	55.6% (36)	83.4 (30–132)	36 (42.4%)	13 (18.3%)
Meningitis	59	16.7	3.9	11 (18.6%)	1 (shunt-related)	73.1	17 (28.8%)	8 (13.6%)	26 (44.1%)	20 (33.9%)	36 (61.0%)	41% (17)	73.0 (40–124)	14 (23.7%)	10 (21.7%)
Hemorrhagic	57	7.0	3.4	11 (19.3%)	0	77.9	25 (43.9%)	14 (24.6%)	26 (45.6%)	16 (28.1%)	33 (57.9%)	50% (24)	79.2 (51–141)	18 (31.6%)	10 (27.0%)
Malformative	55	62.0	2.8	11 (20.0%)	1 (shunt-related)	80.6	27 (49.1%)	15 (27.3%)	19 (34.5%)	18 (32.7%)	20 (36.4%)	71% (17)	88.3 (61–118)	24 (43.6%)	14 (35.0%)
Arachnoid cyst	32	72.0	2.5	5 (15.6%)	0	87.2	18 (56.3%)	11 (34.4%)	6 (18.8%)	7 (21.9%)	13 (40.6%)	60% (15)	90.7 (60–135)	17 (53.1%)	12 (60.0%)
Traumatic	15	79.6	2.3	3 (20.0%)	1 (intercurrent)	67.9	4 (26.7%)	4 (26.7%)	6 (40.0%)	3 (20.0%)	9 (60.0%)	(1)	–	4 (26.7%)	4 (28.6%)
Others^b^	28	24.9	3.0	4 (14.3%)	1 (intercurrent)	78.6	12 (42.9%)	10 (35.7%)	8 (28.6%)	4 (14.3%)	13 (46.4%)	37.5% (8)	78.7 (55–101)	12 (41.4%)	8 (40.0%)
Total	456	55.6	2.7	71 (15.6%)	13 (2.85%)	76.4	147 (32.3%)	82 (18.0%)	212 (46.5%)	105 (23.0%)	217 (47.6%)	54.5% (189)	82.3 (30–141)	189 (41.4%)	115 (33.3%)

^a^Non-evaluable cases are patients who have not completed their studies (whatever these are) and whose social outcome is, therefore, not yet determined

^b^“Others” regroups cases of Dandy–Walker malformations (6), other craniocervical or craniofacial malformations (5), and hydrocephalus of undetermined causes (17)

### Social outcome

Schooling was documented in 446 cases (97.8%): 189 (41.4%) followed a normal, complete curriculum; 16 (3.3%) had a short curriculum in a normal school; 70 (15.4%) required some help at school; 145 (31.8%) required schooling in special institutions; and 26 (5.7%) could not be schooled at all.

**Fig. 7:**
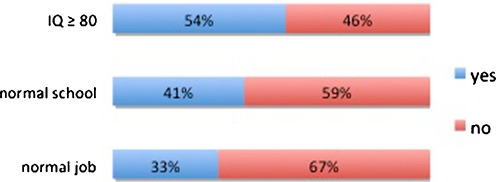
Comparison between IQ testing, schooling, and social integration. Normal IQ was defined as 80 or higher. The discrepancy points at underestimation of handicap by formal testing and academic achievements, representing “the invisible handicap”

Data on the patient’s social outcome were available in 452 patients (99.1%): 111 (24.6%) were still in the course of their studies and were considered not evaluable; 115 (25.4%) had a normal job; 38 (8.4%) were job seekers; 80 (17.7%) were employed on sheltered jobs; and 108 (23.9%) had no occupation. Among evaluable patients, 33.7% had a normal job. Among employable patients (normal job + job seekers), the unemployment rate was 24.8%; as a comparison, the unemployment rate for those under 25 years was 16.3% in 2008 (median of the last control in our study) [12]. In order to illustrate the discrepancy between neuropsychological testing, academic achievement, and social integration, we plotted these variables in Fig. 7.

## Discussion

### Methodology

Our series represents a large number of patients treated during childhood for hydrocephalus and followed up into adult age, based on a relatively stable population sample of four million. Although the accrual of patients was prospective and consecutive, the collection of data for the present study was for a large part retrospective. This leads to some uncertainty, in particular over the real incidence of the different types of sequels, leading to likely underestimation of sequels, but also of the number of pregnant women. The number of patients lost to follow-up is also a limitation: some of them may have died; but more probably, the patients who moved or interrupted follow-up were more able than those who did not. Also, the retrospective evaluation of many patients did not allow the use of more sophisticated scores than the rather crude GOS (5 grades) or KNK autonomy score (11 grades), and neuropsychological testing was available for only a minority of patients. Finally, studying the adult outcome of patients treated at birth amounts to evaluating standards of care of two decades ago. However, these limitations being acknowledged, we think that the present series gives a more realistic view of the outlook of hydrocephalic children than series based on voluntary participation or filling a questionnaire [8, 21, 22]. Obviously, having all patients filling prospectively a form dedicated to hydrocephalus, like the Hydrocephalus Outcome Questionnaire (HOQ) [16, 24], is certainly the gold standard for this type of studies, but this is a huge undertaking and some time will pass before we can see the adult outcome. For the time being, and with the aforementioned caveats, we think our data can be useful.

### Surgical outcome

Shunt malfunction is a permanent risk in patients treated with shunts, carrying risks of both morbidity and mortality. All studies of shunt outcome show similar biphasic survival curves; however, the incidence of very late obstruction is poorly documented in the literature. In a previous study, we found that, after a typical biphasic curve, a shoulder could be seen around 15 years, with a third segment close to the zero line; however, several patients had presented very late with their first shunt malfunction [33]. Our present study confirms that many patients shunted during childhood require reoperation when they are adults, some of them for the first time; it also shows that late malfunction in an unprepared patient can be fatal. Regarding patients treated by endoscopy, the incidence of obstruction is markedly lower than with shunts; however, long-term data are even less clear; whatsoever, it is now certain that delayed obstruction of the stomy is a very serious threat as well [7].

Late shunt infection is a risk inherent in harboring a shunt, and although surgery is the most common source of infection, contamination sometimes comes from other sources. In a previous study, we found that the yearly incidence of infections beyond the first postoperative year was 0.28% [30]. Among the sources of late shunt infection, abdominal complications are prevalent; in particular, the yearly incidence of bowel perforation by the peritoneal catheter was estimated at 0.1%, affecting especially severely handicapped patients [32]. In the present study, shunt infection occurred after the age of 20 in 11 cases and was fatal in 1 case. Patients and their caretakers should be given information on this risk in order to organize prompt diagnosis and treatment.

Evidence of shunt dependence is provided by the occurrence of symptomatic shunt failure. When a patient who has never been revised before presents with an asymptomatic shunt rupture, the question arises of either a ruptured but still functional shunt or an obstructed shunt in a patient who has become at some point shunt-independent. In any case, a broken shunt in an asymptomatic patient should not be taken as a proof of shunt independence, even with a wide gap between the shunt fragments [6]. In our practice, the answer to the question requires a shuntogram, catheter ligation if the shuntogram shows no flow, then shunt removal if the ligature is well tolerated. In the present series, following this procedure resulted in shunt independence in only six cases; in four cases, the test failed to achieve shunt independence. Shunt independence can also be achieved through endoscopy [5]; however, in our series, shunt independence was achieved through endoscopy in only three of the seven attempts. Overall, our experience confirms that, for shunted patients, achieving shunt independence is exceptional; the fact that 22 patients had their first shunt malfunction after 20 years is a reminder that the absence of shunt malfunction does not mean that the shunt is useless. The old dictum “once a shunt, always a shunt” admits very few exceptions.

### Mortality

Few studies focus on the long-term mortality in pediatric patients treated for hydrocephalus. The mortality rate ranges between 13% after 17 years [19], 22% at 20 years [21], and 22% at 10 years [28], but series are often polluted by intercurrent causes of mortality. Focusing on nontumoral hydrocephalus, Tuli and Casey found similar figures (12.6% and 11% mortality at 10 years, respectively) [4, 29]; by contrast, Paulsen found a shunt-related mortality rate of only 2.9% after 20 years [21]. In our series, 6.3% of patients who had survived their 20th birthday died during their third decade, and shunt-related mortality represented two fifth of these. Patients with myelomeningocele were overrepresented in shunt-related mortality; as reported earlier, the excess risk being likely related to the Chiari malformation [29]. Unexpected death in a shunted patient should be considered shunt-related unless proved otherwise [13]. In a previous study, we noted that the only mean at our disposal to prevent such catastrophic outcome was systematic follow-up and revision of asymptomatic ruptured shunts [31]. The periodicity and modalities of this follow-up are open to debate and depend on the medical resources available.

### Functional outcome

Neurological sequels are mostly related to the cause of hydrocephalus rather than hydrocephalus itself, but can also result from the initial severity of hydrocephalus and episodes of acute shunt malfunction. The profile of handicap differs markedly between the different groups, as shown in our Table 1. Motor sequels affect up to 60% of patients [10], and Platenkamp reported that 13% needed aides for walking and 17% needed a wheelchair [24]. Visual sequels include vision loss, oculomotor problems, visual field defects, and visual-perceptive deficiencies, affecting up to 83% of patients according to Andersson [1]; these result from optic nerve atrophy and brainstem and cortical lesions mostly caused by episodes of intracranial hypertension. In our series, 13.6% of adults were recorded as having a significant visual impairment. Endocrine sequels are also common in hydrocephalic patients; in particular, disturbance of the gonadostimulin axis; although precocious puberty is a common finding during childhood [25], its relation to hypothalamic dysfunction and its predictive value for low fertility in adult life are poorly documented. In the series published by Gupta, only 26.2% of adult women answering the survey reported having been pregnant [8], which may still be an overestimate; conversely, our figure (14.5% of female patients) may be an underestimation because of the retrospective bias and relatively young age of our patients. Low fertility is also a reflection of the poor social life and isolation of many of these patients [8]. Epilepsy is common in hydrocephalic patients and is caused by the initial brain disease as well as the insertion of the catheter, as attested by the presence of epileptic foci on postoperative EEG [2]. Piatt found that the incidence of new-onset epilepsy was around 2% per year [23]. In our series, we found similar figures in the early years after surgery; however, new cases of epilepsy became rare as the patients grew up. Epilepsy has been correlated with a lower IQ [10] and has a negative impact on quality of life measured by the HOQ [16]. Many other ailments are underreported but have a major impact in the quality of life, such as depression, with an incidence as high as 43% [8], or chronic headache, present in up to 44% of adults [27].

### Social outcome

Schooling and social integration of hydrocephalic patients are mostly conditioned by their cognitive sequels. These result from congenital malformation or aggression or from the severity of hydrocephalus and subsequent complications. The severity of initial brain damage is considered the most important determinant of intellectual outcome [26], and its impact is maximal in newborns: in neonatal hydrocephalus, Persson found cognitive sequels in 23 of 48 patients and no sequels in only 4 of 48 [22]. Our results (Table 1) confirm that patients treated early in infancy (mostly with posthemorrhagic and postmeningitis hydrocephalus) or patients with widespread brain damage (posttraumatic hydrocephalus) have a poorer intellectual outcome than patients whose hydrocephalus develops later and who have a more focal brain lesion (like tumors or arachnoid cysts). Platenkamp reported normal schooling in 59%, special education in 33%, and no schooling at all in 9% [24]. In our series, the figures (special education in 31.8% and no schooling at all in 5.7%) are similar; the additional categories “short curriculum” and “help at school needed” shed also some light on the specific needs and final academic achievement of these patients.

Generally, cognitive handicaps become more evident as the patients advance in their school curriculum and become adults [11]. In a large study on 403 patients tested after a follow-up of 20 years, Gupta et al. found that 56.3% were functioning normally and 58.8% were fully independent adults [8]; however, this study was based on voluntary participation and suffers a bias of selection. Results in consecutive series show less favorable results: 40% of the patients evaluated by Hoppe-Hirsch et al. had dropped out of normal school curriculum [10]. Among 82 patients followed up beyond the age of 16, Kokkonen found a normal intellectual functioning in 45.8%, schooling was normal in 60%, but only 11% had a normal job [15]. Paulsen et al. found it more meaningful to select patients aged more than 20: 23% of their patients were still studying, 33% were working in the competitive labor market, 23% were on sheltered jobs, and 21% were “not in contact with the labor market due to chronic illness” [21]. We found a similar number of patients still studying (24.6%) and not evaluable regarding social integration; however, our employment rates, both on normal and sheltered jobs (25.4% and 17.7%, respectively), were lower, which can reflect a less favorable socioeconomic system, but also a cohort of patients with a generally more severe illness. Our Fig. 7 illustrates that handicaps are underestimated by formal testing, as well as by school evaluations, and that only few patients pass the final test: social integration in a competitive environment. The highest functions of the brain, which are the most important for social integration, are also the most elusive to testing.

### Child-to-adult transition

There is more or less a consensus over the need to follow-up patients with hydrocephalus during all their childhood. Rare opinions are voiced, challenging the practice of systematic follow-up of hydrocephalus as a misuse of strained neurosurgical resources and proposing to dismiss the patients to pediatricians or general practitioners [14]. However, every now and then, grim stories are published as stark reminders that, without routine and apparently useless follow-up, terrible drama can strike [3, 7]. Despite of this, opinions diverge over what happens when the patients become adults, and no guideline exists for the long-term follow-up. The general view is that the follow-up has to be continued: Casey advised follow-up clinics every second year until the age of 16 [4]; Paulsen proposed a follow-up in adulthood every 4 or 5 years [21]. However, we think that very long intervals between clinics may lead many patients to abandon follow-up altogether. In our experience, many patients are anxious to continue the regular follow-up in neurosurgery, and they grudgingly accept being transferred to an adult colleague by the pediatric neurosurgeon.

Transition from child to adult care has been defined as “a multidimensional, multidisciplinary process that addresses not only the medical needs of adolescents as they move from children’s services to adult services but also their psychosocial, educational, and vocational needs” [20]. This holistic definition sets very high standards, which are rarely met. Regarding hydrocephalus, the patient’s health needs are highly varied, according to the severity of sequels, and our Table 1 shows a number of different profiles according to the cause of hydrocephalus. Knowledge of these different needs is useful in order to anticipate the process of transition. Patients who have no physical impairment need only a neurosurgical follow-up. Some need to be persuaded to continue this follow-up; conversely, others need to be encouraged to live a normal life and convinced that they should not be forbidden any activity because of their shunt. Generally, the follow-up of these patients has to tread a line between too much fear and too much confidence. In these purely neurosurgical patients, pediatric neurosurgeons have a prominent role to play in managing the transition and “apply their expertise to management of the adult patient” [9]. This does not apply to patients with more severe mental handicaps, who need daily care, or to patients with spina bifida, who pose complex problems and are best managed by rehabilitation specialists [33]. In any case, education of the patient and his family requires collaboration, anticipation, and planning because transition is “a process, not an event” [18].

## Conclusions

Most children treated for hydrocephalus now reach adult age; however, data on their outcome are still rare and controversial. Our series, in spite of its biases, gives a picture of what these patients have become and which problems they face in adult life. These patients present with a wide spectrum of ailments, with an impact on autonomy ranging from crippled to fully autonomous person. Our study also shows that the incidence of shunt malfunction, potentially fatal, becomes low but never null in adulthood. A life-long neurosurgical follow-up of these patients must be organized and adapted to the specific needs of each patient.
